# Men’s Sheds in Scotland: the potential for improving the health of men

**DOI:** 10.1057/s41271-020-00268-5

**Published:** 2021-02-04

**Authors:** Danielle Kelly, Simon Teasdale, Artur Steiner, Helen Mason

**Affiliations:** Yunus Centre for Social Business and Health, Glasgow Caledonian University, M201 George Moore Building, Cowcaddens Road, Glasgow, G4 0BA UK

**Keywords:** Men’s Shed, Health, Public health

## Abstract

**Abstract:**

Recent policy focus is on the ‘non-obvious’ role of community-based organisations in tackling causes of poor health, such as social exclusion. Men’s Sheds are a type of community-based organisation offering health and wellbeing benefits to men, despite this not being the explicit reason they exist. A qualitative study was conducted in Scotland to identify sustainability challenges that impact on the ability of Sheds to become a formal healthcare service. Findings showed that a reliance on ageing and retired volunteers to undertake operational tasks and generate income to fund activities affected the ability of Sheds to sustain and develop. Further, members preferred their Sheds to remain informal and flexible to fit their specific needs. Although Sheds are recognised for their health and wellbeing benefits to men, policymakers must recognise that formalising their activities might detract from the Shed’s primary aims. This paper summarises specific policy implications and recommendations, taking into consideration tensions between the expectations placed on Sheds to expand into formal healthcare delivery, and the needs of Shed users.

**Highlights:**

- Men’s Shed are known for their ability to contribute to men’s health and wellbeing.
- The potential of Shed to take on a formal healthcare role is questionable because of a reliance on ageing and retired volunteers and a preference to remain informal.
- Policymakers must recognise that although Sheds might be well placed to offer formal health care this may detract from their primary aims.

## Introduction

Over the past few decades, policy and research has shifted focus to the social determinants of poor health rather than purely the consequences [1–3]. Growing research has called for a recognition of the contributions of community-based organisations in addressing social determinants, such as isolation and social exclusion [4–6]. Further, there have been calls from the non-profit sector for the channelling of public health funds from Government and national health authorities to expand the scope and remit of such organisations to impact on the health of communities on a larger scale [4]. Research has shown that community-based organisations tackle ‘causes of the causes’ of poor health by fostering local social networks and inclusion, employment and educational opportunities [6, 7]. However, many organisations may not recognize the contributions to improving health they may provide, nor state health improvement among their objectives. Further, causal pathways linking community activity to improved health and wellbeing are not easy to identify [8]. In many cases, positive health benefits come from activities that organisations offer, rather than from providing direct health-related services [9]; for example, community cafes or transport aimed at connecting social isolated populations [10]. The long-term sustainability of these types of community-based organisations can also be affected by insecure funding and reliance on volunteers with limited commitment, skills, or capacity to maintain their activities [11, 12].

Men’s Sheds (Sheds) are a type of community-based organisation recognised for their potential contribution to the health and wellbeing of men [8, 13]. Sheds first emerged in Australia in the 1990s amidst concerns about de-industrialisation and growing numbers of men isolated and socially excluded through unemployment. Since that time, Sheds have expanded internationally, especially in ‘western’ countries such as UK, Denmark and Ireland. They offer participation in activities such as woodwork and gardening, and also opportunities for men to socialise [14]. Few studies have explored Sheds’ tackling of health issues, but some show that social participation and meeting new people in Sheds can improve mental health and social wellbeing by giving men a sense of purpose [15, 16], and through reducing social isolation and depression [17, 18]. Moreover, Sheds have been recognised for their ability to attract ‘hard to reach’ men who are unlikely to engage in any type of formal health care [19].

Sheds have received international Government attention, with the Federal Department of Health in Australia targeting funds to broaden Shed services and support men’s health [20], and the Scottish Government highlighting Sheds’ tackling of social isolation and loneliness [21]. In Scotland, the setting for this study, Sheds have developed quickly, from the first Shed opening in 2013, to approximately 180 registered with the Scottish Men’s Shed Association in 2020. Although Sheds are informal community organisations, research has highlighted the potential of Sheds to become formal health care providers with directed support from government and health authorities [22, 23]. However, wider voluntary sector literature has suggested that engaging community-based organisations in formal healthcare delivery may run contrary to their values [24, 25].

We explore what issues affect the sustainability of Sheds, and from this discuss the feasibility of Sheds to deliver any kind of formal health care with a more explicit health agenda in Scotland. We identify four key sustainability challenges and discuss ways to tackle them, taking into consideration that the delivery of health services by Sheds might detract from the fundamental characteristics that attract the men to participate; the ‘ethos’ of Sheds. If Sheds are to contribute to improving the health of their users, on a basic level, they must be able to sustain and survive. Further, organisations themselves will need to determine their priorities to ensure that the appeal of attending a Shed is not weakened if Sheds take on a more specific formal health role. For this reason, policymakers must be aware, through research, of what Sheds can reasonably be expected to deliver. Thus we set out to gather views and opinions from Shed members and other interested parties to enhance understanding of sustainability challenges for Sheds, and their suitability for providing formal healthcare services.

## Data and methods

We conducted a qualitative study in which we interviewed Shed members, individually and in groups, and a range of key Shed stakeholders including members of regional and national Shed Associations, representatives from the Scottish Government, and support agencies from the non-profit sector.

### Recruitment

The sampling frame for this study consisted of all Sheds registered with the Scottish Men’s Shed Association (SMSA)–a national charity organisation that supports Sheds. At the time of recruitment (January 2018), the SMSA had registered 98 Sheds. We included Shed’s with: a minimum of 20 members; a fixed-space for members to meet and for data collection; and regular, no less than fortnightly, activities (so members could comment meaningfully on sustainability challenges). Fifteen Sheds met the criteria; we selected five to ensure geographic and demographic variation, both rural and urban, and from low and high resource areas [26]. The study team contacted the five Sheds by telephone, email, or visited to assess willingness and availability of members to participate. We then used snowball and convenience sampling techniques to recruit Shed members to interview [27].

The SMSA provided a contact list of key Shed stakeholders, defined by the study team as non-Shed members engaged in the sustainability and development of Sheds in Scotland: members of the national and regional Shed Associations, development officers from the non-profit sector, Scottish Government representatives, and community development officers working for the state. Working from this list we contacted key Shed stakeholders by email or phone to explain the research and invite them to take part.

### Data collection

We conducted semi-structured interviews with 62 Shed members and six other stake holders between 1st April and 1st December 2018. We developed a topic guide of issues about Shed sustainability, which focused on factors that may affect Sheds’ survival and capacity to deliver regular activities (See Box 1). The questions addressed economic and social sustainability, types of support available for Sheds, with leeway for interpretation and flexibility to discover relevant new themes [28]. We also asked participants about the health concerns prevalent within their Shed and wider community as background for considering the potential for the Shed to provide a formal health care service.

We conducted interviews with Shed members on Shed premises in a quiet area. We arranged group interviews for Shed members who preferred this to individual interviews [29]. We interviewed key Shed stakeholders at their workplaces, or at the researcher’s institution. We gave all participants information sheets that explained the nature of the study and all of them completed consent forms.

We conducted 68 interviews of 30–60 min, audio-recorded and transcribed them, and analysed the qualitative data using NVivo software. The details of the five Sheds that took part in the study are shown in Table 1. The total of 68 participants interviewed included six key Shed stakeholders, 23 Shed committee members, and 39 non-committee Shed members. The mean age of the Shed members was 69 years.

**Table 1:** Study participant description

	Shed characteristics	Number of participants interviewed	Shed activities
Shed 1	100 + members Urban location One of largest Sheds in Scotland Leasing premises Operates weekdays	5 Committee members 7 Non-committee members	Woodwork, metalwork, leatherwork, games, social events, educational visitors
Shed 2	30 + members Remote rural location Affluent area Weekly rental of community hall Operates weekdays	6 Committee members 5 Non-committee members	Choir, social events, educational visitors, games
Shed 3	80 + members Urban location Mixed gender Leasing premises Operates weekdays	5 Committee members 10 Non-committee members	Woodwork, metalwork, painting, computing, musical instruments, social events, educational visitors
Shed 4	20 + members Urban location Deprived area Set up by local authority Leasing premises Operates weekdays	3 Committee members 7 Non-committee members	Woodwork, bike renovation, social events, gardening
Shed 5	20 + members Urban location Based in a school (does not have any overheads like rent or bills) Operates evenings and weekends	4 Committee members 10 Non-committee members	Woodwork, metalwork, social events, educational visitors

**Box 1:** Shed topic guide

1. Can you tell me a little bit about yourself, your role and your affiliation with the shed?	
2. Can you tell me a little bit about your shed?	
What is the purpose of the shed?	
What do you think it does well?	
How important is it to the members/community?	
3. Does your shed have a strategy or action plan for the future? If so, can you tell me about this?	
What are your main areas for growth/ development?	
Where do you see yourselves in 5–10 years’ time?	
4. What does the term sustainability mean to you? What does the term sustainability mean to you?	
5. What do you think are the main challenges for the sustainability of your shed? Why?	
Economic? Social? Other?	
6. What do you think might help your shed be more sustainable?	
Is there anything else you could be doing?	
Are there any barriers to this?	
7. Have you received any help from external organisations or bodies to be more sustainable? If so, can you tell me about this?	
8. Do you think there might be any barriers to implementing entrepreneurial changes through the processes involved in this project?	

We used descriptive coding techniques to identify relevant information and topics. We merged duplicate codes and categorised data under larger headings and subheadings [30]. We focused on emergent themes of Shed sustainability challenges. We used content analysis to ascertain the frequency of mentions of specific topics related to Shed sustainability. The research team discussed the process throughout, gathering feedback and seeking consensus. The host University granted ethical approval for the study.

## Results

Sheds in this study served predominantly older and retired men, often with health issues related to ageing, and a lack of motivation to take part in ‘work-like’ activities that replicated previous employment (See Table 1 for activities). Health concerns faced by Shed members included poor physical and mental health, substance use, and health problems related to social exclusion, isolation and unemployment. Sheds 1, 3 and 4 included members of 30-50 years of age who were unable to work due to long-term physical and mental health issues. All Sheds shared a specific ‘ethos’ (a term coined by Shed members and stakeholders): the organisations were run ‘by the men for the men’ and did not oblige members to take part in any set activity or agenda. The highest priority for all were the members’ leisure needs (quote 1-See Table 2 for all quotes).

**Table 2:** Quotes

Quote number	Quote
1	*‘The Shed is whatever you want it to be, it’s an important space for men to get together and be themselves and relax. Whether there’s an output at the end of it? It doesn’t really matter’ (State community development officer).*
2	*‘We can’t just open, we need supervisors, we need key holders, we need first aiders here, and therefore it’s a commitment...but the supervisors are just volunteers’ (Shed committee member).*
3	*‘I came down here to relax in my retirement. Don’t get me wrong, the Shed has changed my life, I’m involved in a lot, but I’m taking on too much it’s getting a bit stressful’ (Shed member).*
4	*‘At any time we could disappear, there needs to be someone else who knows what they’re doing, that’s what happens when you get to our age, you go over on your ankle and that’s it, or take the flu...we are not going to be here forever’ (Shed committee member).*
5	*‘It’s hard to talk to the shedders without feeling like you are managing them or bossing them around…shedders might think this way is too bureaucratic’ (Shed committee member).*
6	*‘We are here for ourselves, we can’t get a chance to get on the machines if we are too busy looking out for other people, we don’t have the expertise’ (Shed member)*
7	*‘Social work or occupational health might think “Well, you can bring somebody down here for two hours and leave them”, because it’s not the men’s responsibility to care for somebody, they can’t’ (State community development officer).*
8	*‘We care about people, there’s people in here living with dementia...but safety is their responsibility, so if they go to machine and can’t use it, they don’t use it’ (Shed member).*
9	*'We need to be near transport links, if you were further out with the area you’ve got to use your car, so older people will be relying on a lift, you’ve got to be able to get here by public transport’ (Shed member).*
10	*The biggest issue is that the council departments are not understanding the health benefit (in giving Shed premises)…To be fair to them, they’ve never seen this model before. So, there’s got to be this whole education thing that we try to do’ (Shed Association member).*
11	*‘...we have loads of Sheds (in Scotland) that have short-term low cost rental agreements that might last a few years. But what is going to happen in a few years when this stops or councils need to make some money so increase rents?’ (Shed Association member).*
12	*Paying the rent is the main challenge...I would hate to see the Shed close because we can’t afford to keep it running’ (Shed committee member)*.
13	*‘It’s time consuming (making items to sell), people want to do other things, they’ve been putting themselves under pressure, everybody’s coming down here to relax, they didn’t sign up for this’ (Shed member).*
14	*‘At the moment…the funding’s very much small pockets…Sheds will need to get more creative and evidence their impact more effectively as that funding pot gets smaller…they’ll have to compete for that funding’ (Non-profit development officer)*.

When asked about their own Sheds, participants stated that they did not view their Shed as a formal service of any kind, but that they did explicitly aim to provide a practical and safe space for community members of all backgrounds to meet. Committee members from three of the five Sheds all agreed that the general improvement of the health and wellbeing of their members was a secondary aim, and that these Sheds did not target specific health outcomes. All of the interview participants from all five Sheds (62 members) reported that they did not want the Shed viewed as offering a formal ‘service’ or as a health provider. They preferred that their Sheds remain an informal and unstructured space for men to meet in their communities whenever they wished. For these reasons, those interviewed said that they avoided formalising their activities or operations, aiming to protect the informal and flexible nature of their organisation and the immediate needs of their members.

We found four key areas that may adversely affect their sustainability and the potential to play a role in improving the health of older men:

- Recruiting volunteers to undertake operational tasks;
- Dealing with members whose heath needs are complex, including negotiating responsibility for care;
- Acquiring premises suitable for activities to meet member needs;
- Generating income to fund Shed activities.

Other challenges including recruiting new members (including advertising) and ensuring adequate communication among members (telephone and email contact) but analysis of this is beyond the scope of this paper.

### Recruiting volunteers to undertake tasks

All of the Sheds required volunteers to contribute to the day to day running of the organisation. Like other voluntary organisations, each Shed depended on a committed management committee to acquire premises and find funding to ensure survival. A small committee of volunteers (including chairperson, treasurer and secretary) completed administrative tasks. Sheds also required other members to run activities and events or perform tasks such as training members how to use tools and equipment (quote 2).

Core committee members often felt overburdened; their administrative roles added pressure and stress in their lives (quote 3). Shed 4 struggled to recruit committee members as a high proportion grappled with health issues that limited their participation. As most of the members of all Sheds studied were 60 years of age or over, committees risked losing members to age related ill-health or death, increasing strain on those members who remained (quote 4).

Many retired members reported disinterest in assisting the committee or engaging in ‘work-like’ tasks that mimicked their previous employment; they attended for their own needs and to escape responsibility. Committees members reported they did not want to pressure members or diminish their enjoyment (quote 5). Sheds interviewees reported that the lack of volunteers for administrative activities meant they worried about long-term sustainability. They had little capacity to recruit new members or expand activities to meet the health and wellbeing needs of their community.

### Dealing with complex health needs

The Sheds in this study did not provide formal healthcare for those with physical or mental health issues, for example depression. None of the Shed members interviewed had any formal training qualifications for dealing with people with complex health issues. Shed volunteers interviewed found it difficult to accommodate individuals with complex physical and mental health needs; they reported that care for others exceeded what they felt prepared to do, and that it was not their responsibility (quote 6).

Shed committee members reported that they tried to avoid taking on such complex cases. Turning people away, however, ran counter to their inclusive ethos. Administrative committees grappled with decisions about including men referred to their Sheds from state health services, such as doctors or care workers (quote 7).

Shed 4 allowed those with complex health needs to participate if accompanied by a registered social worker or carer (including paid carers or family members). However, this led to further referrals from state health services that the Shed could not accommodate. Members questioned the safety of those with complex health needs working with potentially dangerous tools and machinery (See Table 1), and Shed liability should an accident occur (quote 8). Members from Sheds 1 and 3 also expressed concerns over monitoring the mental and physical deterioration of their members, especially older members showing signs of Dementia and Alzheimer’s. These findings showed a tension between the demands of public health professionals and the desires of Shed members to protect their ethos.

### Acquisition of premises

Acquiring and sustaining adequate premises for Shed activities challenged all Sheds. Respondents explained the importance of reaching men across their communities, and the prime role of location and accessibility, especially for accommodating less mobile older members (quote 9).

Volunteers lacked skills and knowledge to acquire premises. Shed 5 had secured access to a local school woodwork department, but with restricted hours for their members to use it. The other four Sheds struggled to gain full ownership of buildings or land, or to secure long-term, low-cost rental agreements from private landlords or public sector authorities. Key Shed stakeholders reported reluctance from public sector authorities to pass ownership of buildings and land to Sheds because of authorities’ lack of understanding of what Sheds do, including their potential benefits to health and wellbeing (quote 10).

Premises available for Sheds were often unsuitable due to a lack of space, high costs for building refurbishment, uncertainties with planning permissions, or the instability of lease agreements (quote 11). Sheds 3 and 4 had lease agreements with their local councils, however, the lease agreement of Shed 3 did not allow for any modifications of the building to accommodate increasing numbers of members. Shed 4 encountered damp and poorly ventilated premises. With predominantly older members, Sheds were not able to maintain the building themselves, causing further challenges to their sustainability.

### Funding

Sheds often faced difficulties paying rental costs and utility bills, and sourcing funding to grow their Shed and expand activities (quote 12). All Shed respondents placed value on leisure activities that encouraged socialisation amongst members as a way to address issues like social isolation and exclusion. Therefore, they could not pressure members to spend their social and leisure time generating income. As a result, Sheds often relied on funding from external sources, such as community donations and charity grant funding. Shed 4 operated in an area of low income and high unemployment rates, therefore members were less able to contribute their own money, and their Shed could not gather donations in their local community. Financial pressures led four of the five Sheds to produce items to sell at local events, and to offer a paid repair service in their community (such as fixing park benches or making planters). This generated a small amount of income, but also placed unwanted obligations on members. Thus, fundraising often conflicted with members’ preferred characteristics of Sheds as places for relaxation (quote 13). Applying for external grants to sustain Shed activity could be very competitive and members often did not possess skills and knowledge to fill in applications. High competition for funding often led to a prevailing *any money is good money* (Shed member) attitude, detracting members from the activities they aimed to deliver. Further, Sheds are relatively unknown and funders have yet to be persuaded of their benefits, especially those for health and wellbeing (quote 14).

## Discussion

Research has called for increased recognition of community-based organisations, and the channelling of public health funds from government and national health authorities to expand their scope and contribution to addressing social determinants of health on a larger scale [4–6]. Sheds provide practical and social activities to meet immediate needs of men in communities, especially those with physical and mental health issues. Sheds may be well placed to engage in formal health care delivery to meet challenges such as social exclusion. Our study has shown, however, that their sustainability and ability to contribute to this agenda is questionable. Although our study was exploratory, small scale, and confined to Scotland, the four key challenges for Shed sustainability likely apply to Sheds (and other informal community-based organisations) internationally.

Three of the challenges we have identified relate directly to Sheds’ ability to acquire resources (volunteers, premises, income), and are familiar to many community-based organisations [31, 32]. The challenge of dealing with complex health needs suggests that placing expectations on Sheds to expand their activities into formal health care is in conflict with their ‘ethos’ of minimizing obligations and responsibilities for members. Our paper shows that channelling public health funding to Sheds would be problematic as it would require them to change how they operate. Doing so may produce negative unintended consequences as the unique informal characteristics of Sheds and their local presence in communities is the very reason for men’s engagement in activities that may improve their health and wellbeing [8].

## Conclusion and recommendations

MacKinnon and Derrickson [25] point to a tension between ‘resilience’ and ‘resourcefulness’ that is faced by Sheds. Resourcefulness implies a need to protect the Sheds’ ethos, and ‘making do’ with the limited resources available to them. Resilience implies adapting what they do to conform to become a formal public health service. Thus, policymakers and researchers to be aware of such tensions when seeking to draw Sheds into more formal roles. The community development field has long-recognised the difficulties in seeking to provide policy support to ‘bottom-up’, community-based initiatives [11, 24]. Such lessons could usefully be applied to Sheds in aiming to increase the number of such organisations in communities, rather than expanding the capacity of existing Sheds. This study shows that although Sheds are unlikely to have the capacity or want to provide a formal health care service to men in communities, they could still provide a complementary informal route for male health improvement. Especially for ‘hard to reach’ men who might not otherwise engage with any other type of health care [19]. With this in mind, policymakers need to consider ways to support the sustainability of Sheds to enable them to continue to provide informal activities that improve men’s health and wellbeing in ‘non-obvious’ ways. This could be in the form of funding streams to cover core costs such as rent, funding to pay volunteers taking part in key administrative and operational tasks, and access to support and guidance that allows them to support members with complex health needs. As this study focused primarily on the key sustainability challenges facing Sheds, further research is required to clarify how exactly Sheds (and other similar community-based organisations) may fit into healthcare agendas and in what capacity. To some extent this requires a new mind-set for policy makers and public health professionals to find novel ways of engaging with these types of small-scale informal community organisations, and ways of channelling resources to support their existing contributions rather than seeking to responsibilize them into more formal health care roles.
